# Primary Multidrug-Resistant Leprosy, United States

**DOI:** 10.3201/eid1901.120864

**Published:** 2013-01

**Authors:** Diana Lynn Williams, Timothy Hagino, Rahul Sharma, David Scollard

**Affiliations:** Author affiliations Health Resources and Services Administration, Baton Rouge, Louisiana, USA (D.L. Williams, R. Sharma, D. Scollard);; Louisiana State University, Baton Rouge (D.L. Williams, R. Sharma, D. Scollard);; John A. Burns School of Medicine, Aiea, Hawaii, USA (T. Hagino)

**Keywords:** Leprosy, drug resistant, primary, multiple drug resistant, molecular drug resistance testing, SNP, VNTR, molecular typing, Mycobacterium leprae, United States, tuberculosis and other mycobacteria, antimicrobial resistance

**To the Editor:** Since the initiation of multidrug therapy for leprosy (Hansen disease) in the 1980s by using rifampin, dapsone, and clofazimine, resistance to rifampin and dapsone has been observed worldwide and is still prevalent (*1*,*2*). Because few alternative effective antileprosy drugs exist, resistance to these first-line drugs could seriously affect leprosy control programs. We report a documented case of primary multidrug-resistant (MDR) leprosy in the United States.

A man from American Samoa migrated to Hawaii at age 25 years and, at age 41 years, first sought care for generalized erythematous papules and plaques. A skin biopsy showed borderline lepromatous (BL) leprosy (Figure, panel A). He had no prior history of leprosy and no prior treatment. He was treated for 44 months with a daily regimen of dapsone (100 mg), clofazimine (100 mg), and rifampin (600 mg). He appeared to comply with this regimen, and the lesions slowly resolved. He remained free of any new lesions until 4 years after completing treatment, when multiple brown hyperpigmented patches appeared on his lower legs. A skin biopsy showed only hemosiderin deposition but no organisms.

**Figure:**
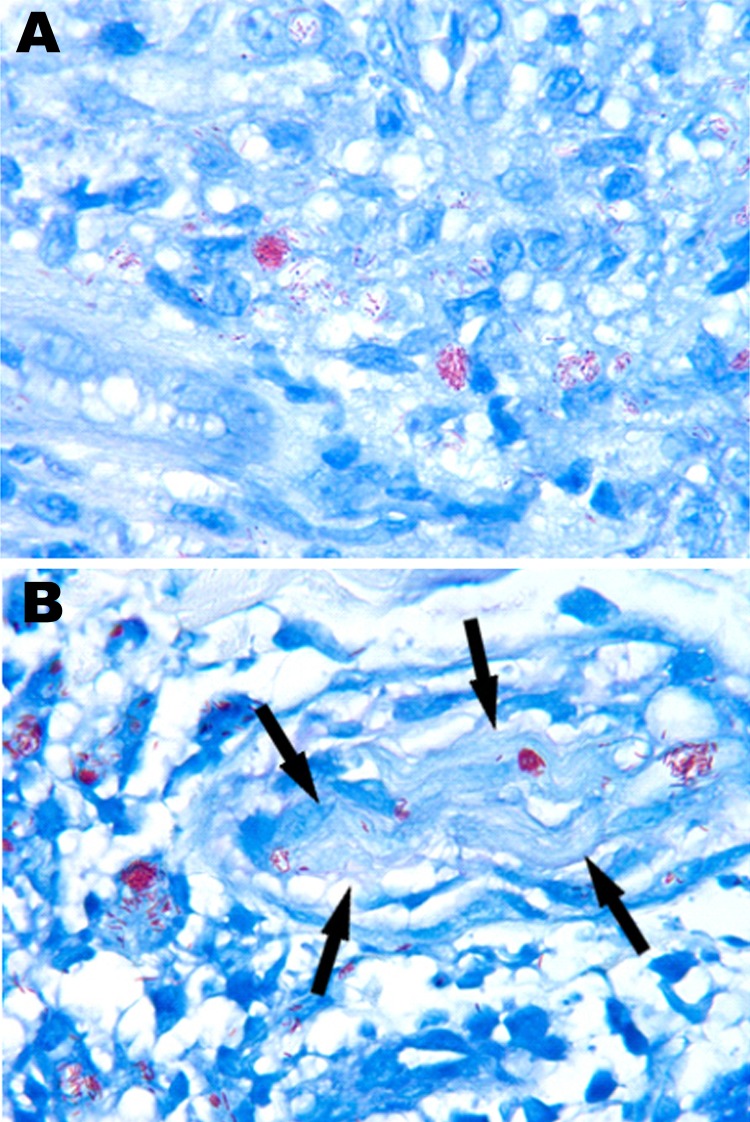
Acid-fast organisms from biopsy specimens of a man with leprosy, United States. Fite-stained sections show numerous acid-fast bacilli in the initial skin biopsy specimen (A) and in the biopsy specimen taken at relapse, 6 years after completion of treatment (B). Both specimens demonstrate the clumps of *Mycobacterium leprae* referred to as globi. In panel B, bacilli can be seen within a cutaneous nerve (arrows), a finding that is pathognomonic of *M. leprae*. Original magnification ×1,000.

At 51 years of age, 6 years after completing treatment, the man again sought care for a 2-week history of multiple generalized erythematous papules and plaques on his face, trunk, and extremities. Some lesions were pruritic but nontender. A skin biopsy showed chronic inflammatory infiltrates with numerous acid-fast bacilli (Figure, panel B). Clinically considered to have relapsed BL leprosy, he was again treated daily with dapsone (100 mg), clofazimine (50 mg), and rifampin (600 mg). After 1 month of this regimen, no clinical improvement was observed.

Real-time PCR using the *Mycobacterium leprae*–specific repetitive element assay (*3*) confirmed the presence of *M. leprae* in biopsy specimens taken at the initial diagnosis and at relapse. Molecular genotyping of these samples with a panel of single-nucleotide polymorphism (SNP) and variable number of tandem repeat (VNTR) markers (*4*) showed that both biopsy specimens harbored *M. leprae* with the identical SNP subtype 3I and VNTR profile. PCR/DNA sequencing of the drug resistance–determining regions of *M. leprae* from these samples showed mutations within codon 53 of the *folP1* gene (ACC→GCC) and in codon 425 of the *rpoB* gene (TCG→TTG). These mutations have been characterized to induce high-level resistance to dapsone and rifampin, respectively (*5*,*6*). Careful evaluation of electropherograms of these drug resistance–determining regions showed only the resistant alleles in both strains.

These data indicated that this patient had been infected with MDR *M. leprae* before his initial treatment for leprosy. Therefore, when he was initially treated with leprosy multidrug therapy, he was essentially given clofazimine monotherapy. This treatment appears to have resulted in a slow, temporary clinical improvement. After relapse, he was placed on a daily regimen of clofazimine (100 mg), clarithromycin XL (500 mg), and minocycline (100 mg). The lesions clinically improved within 2 weeks, and the patient no longer noted any pruritus or tenderness in the lesions.

This report documents a case of primary MDR leprosy in the United States. In evaluating several previous biopsy samples from other patients in Hawaii, we have not seen any rifampin-resistant or MDR isolates. Health officials in American Samoa, the patient’s country of origin, indicated that they were not aware of drug-resistant *M. leprae* among their patients (D. Scollard, pers. comm.). The patient reported no family history of leprosy, and no other contact could be identified. The origin of the MDR *M. leprae* in this case cannot be definitively determined.

Drug-resistant leprosy, including dapsone- and rifampin-resistant and MDR leprosy, has been reported in other parts of the world, usually in association with relapse after insufficient therapy (*1*,*2*). Relapses in leprosy are not usually seen until many years after completion of treatment (*7*,*8*). In the United States, among patients treated for 2 years with a multidrug protocol involving daily rifampin, no relapses were observed after 10–15 years’ follow-up (*9*). Most new or worsening skin lesions clinically suspected to be relapses are actually leprosy reactions (*10*), which affect 30%–50% of patients. In the patient reported here, leprosy relapsed with MDR *M. leprae* 6 years after completion of treatment.

The emergence of drug resistance poses a serious threat to leprosy control programs that rely on a secondary intervention, such as chemotherapy, because a leprosy vaccine is not available. Clinicians should be aware that persons who have acquired leprosy in other countries could have infection resulting from drug-resistant *M. leprae*. When resistance is suspected, biopsy samples should be analyzed by using molecular assays that enable rapid identification of mutations associated with drug resistance directly from paraffin-embedded biopsy specimens. For patients in the United States, this analysis is available through the National Hansen’s Disease (Leprosy) Program (www.hrsa.gov/hansensdisease/diagnosis/index.html), and for US patients, the program provides the 3-drug regimen for leprosy free of charge. When needed, minocycline, clarithromycin, and ofloxacin are provided as alternatives to treat leprosy.
